# Infant mortality in Brazil, 1980-2000: A spatial panel data analysis

**DOI:** 10.1186/1471-2458-12-181

**Published:** 2012-03-12

**Authors:** Ana Maria Barufi, Eduardo Haddad, Antonio Paez

**Affiliations:** 1 Department of Economics, University of São Paulo, São Paulo, Brazil; 2 Department of Economics, University of São Paulo, CNPq scholar, São Paulo, Brazil; 3 School of Geography and Earth Sciences, McMaster University, Hamilton, Ontario, Canada

## Abstract

**Background:**

Infant mortality is an important measure of human development, related to the level of welfare of a society. In order to inform public policy, various studies have tried to identify the factors that influence, at an aggregated level, infant mortality. The objective of this paper is to analyze the regional pattern of infant mortality in Brazil, evaluating the effect of infrastructure, socio-economic, and demographic variables to understand its distribution across the country.

**Methods:**

Regressions including socio-economic and living conditions variables are conducted in a structure of panel data. More specifically, a spatial panel data model with fixed effects and a spatial error autocorrelation structure is used to help to solve spatial dependence problems. The use of a spatial modeling approach takes into account the potential presence of spillovers between neighboring spatial units. The spatial units considered are Minimum Comparable Areas, defined to provide a consistent definition across Census years. Data are drawn from the 1980, 1991 and 2000 Census of Brazil, and from data collected by the Ministry of Health (DATASUS). In order to identify the influence of health care infrastructure, variables related to the number of public and private hospitals are included.

**Results:**

The results indicate that the panel model with spatial effects provides the best fit to the data. The analysis confirms that the provision of health care infrastructure and social policy measures (e.g. improving education attainment) are linked to reduced rates of infant mortality. An original finding concerns the role of spatial effects in the analysis of IMR. Spillover effects associated with health infrastructure and water and sanitation facilities imply that there are regional benefits beyond the unit of analysis.

**Conclusions:**

A spatial modeling approach is important to produce reliable estimates in the analysis of panel IMR data. Substantively, this paper contributes to our understanding of the physical and social factors that influence IMR in the case of a developing country.

## Background

Reducing infant mortality rates (IMR) is an important target for Brazil within the framework of the Millenium Development Goals [1]. While generally the emergence of a consensus on the multidimensional character of development can be observed,^1 ^the specific strategy to obtain better developmental outcomes remains controversial [2]. Two broad perspectives have been propounded. First, there is a growth-oriented vision, in which the promotion of economic or income growth can maximize welfare as measured by social indicators (see inter alia [3] and [4]). An alternative vision accepts the connection between income and health outcomes, but argues that the latter may be at least partially affected by mediating variables of a social and environmental nature. Given the policy relevance of the arguments, it is important to assess the impact of various factors on developmental outcomes.

The objective of this paper is to study the determinants of IMR in Brazilian municipalities in the last decades. In particular, we seek to assess for this period, the impact on IMR of economic factors, as well as social factors, health infrastructure, and living conditions. Previous research has studied infant mortality in Brazil, however considering sub-regions of the country (a city or some states, e.g. [5,6]) or more limited temporal scopes (e.g. [7,8]). In this paper, we analyze all municipalities in the country during a period of two decades, from 1980 to 2000, and thus contribute to generate a broader knowledge base regarding IMR in Brazil. Adoption of a spatial modeling approach allows us to consider, in addition to usual variables in the analysis of IMR, the possibility of spatial effects, i.e., spatial autocorrelation which may affect inference, and the presence of geographical spillover effects.

The paper is organized as follows. Previous work in this area is reviewed, before describing the methods and data used in this research. Next, the results of the analysis are presented and discussed. Finally, the implications of the findings are highlighted in the closing remarks.

### Infant mortality in Brazil in review

A number of studies that investigate infant mortality in Brazil are found in the literature. Some of these studies were motivated by the need to evaluate, taking into account socio-economic factors, specific health programs such as the Health Family Program (*Programa Saúde da Família*) and the Community Health Agents Program that were created in a context of decentralization of health services. Peixoto [6], for instance, identifies a significant effect of both programs over the reduction of the IMR in the municipalities of the Southeast of Brazil, from 1999 to 2003. Guanais and Macinko [7] find similar positive effects of these programs between 1998 and 2006, in an analysis of Brazilian municipalities with more than 10,000 people.

A key interest has been on the direct effect of household income on children's health. This is the case of Reis and Crespo [9], who evaluate the impact of income over various health indicators. Controlling for family and individual characteristics, they find that children who live in poorer households are likely to have worse health status than children in more affluent households. Szwarcwald et al. [5] consider census tracts in neighborhoods in the city of Rio de Janeiro, and use a heterogeneous index of poverty concentration to show that the health status of the population (measured by infant mortality-related variables) tends to deteriorate with increasing levels of concentration of poverty. The role of education has also been explored, for instance by Rosenberg et al. [10], in a study that stresses the role of education in the reduction of infant mortality, while controlling for the influence of climate change and regional effects. Thomas et al. [11] also find that parental education has a direct effect over child survival, independently of the link provided by income.

Other demographic factors previously studied include the role of family planning as one determinant of IMR. In this regard, Victora and Barros [12] find that, after considering other socio-economic factors, there is no clear effect of adolescent fertility on IMR. This dimension may synthesize other characteristics of the family where the child was born, such as the level of social support available to the parents.

Given important variations between regions, the geography of infant mortality has also emerged as a topic of interest. For instance, in an exploratory framework, Silva et al. [13] apply Bayesian techniques to map smoothed estimates of infant mortality in Rio Grande do Sul between 2001 and 2004. This approach is useful to control the high instability displayed by IMR in neighboring areas. Bezerra-Filho et al. [8] investigate possible risk factors through the comparison of the spatial pattern of IMR and other relevant variables in the state of Ceará, from 2000 to 2002. A key consideration when working with spatial data is the possibility of estimation and interpretation issues caused by spatial effects [14]. Indeed, the models that Bezerra-Filho et al. [8] estimated to explain IMR display spatial dependency in the error terms, even after the inclusion of socio-economic, demographic and health care variables. Similarly, Seabra et al. [15] apply a spatial error model to explain the regional differences of infant mortality rates in 2000 among MCAs in Brazil. They include the IMR of these areas in 1991 and other observed characteristics in 2000 (per capita income, water access, sanitation, illiteracy rate, urban population) as exogenous variables. Their findings suggest that illiteracy rate is the main factor to explain the IMR level. It is also noticeable that water and sanitation are not significantly relevant. Further evidence of spatial effects (in particular spatial autocorrelation) is revealed for specific regions of the country by exploratory spatial data analysis reported by Victora et al. [16] and Leal and Szwarcwald [17].

Many studies have been conducted using cross-sectional approaches. When data are available for multiple time periods, panel data structures are more appropriate to control for potential time invariant confounders, as for example the systematic underreporting of infant deaths or an upward bias related to regional specific characteristics [12]. A number of studies are based on panel data structures, which allow them to capture all unobserved time-invariant factors that might affect the dependent variable. For instance, geographical and cultural features can vary widely among municipalities and may have a significant effect over health habits.

Alves and Belluzzo [18], for instance, define a health production function (proposed by Becker and Lewis [19]), and find, for the period between 1970 and 2000, that education, sanitary services and higher per capita income are associated with lower infant mortality at the level of municipalities. These authors highlight the role of education as one of the most important factors, based on a variety of panel data models, including an endogeneity control [20]. Gamper-Rabindran et al. [21], on the other hand, examine the effect of piped water on the reduction of IMR at the municipal level in the period 1970-2000. These authors adopt a quantile panel data approach, and conclude that there is a stronger impact of piped water provision over the IMR in poorer and underprovided places than in richer ones. The advantage of such structure is that the authors can use quantile estimation and simultaneously control for time invariant unobservable characteristics of the unit of analysis.

While the studies by Alves and Belluzzo and Gamper-Rabindran et al. benefit from broader geographical and temporal coverage provided by panel structures, as well as greater control over unobservable characteristics, neither of them considers the possibility of spatial effects that may affect estimation and inference. Simultaneous treatment of spatial and temporal effects appears to have received attention in the literature only in recent times. Congdon and Southall [22], for instance, propose Poisson panel models with spatial and temporal dependence in the error term to identify gradients of the IMR among different categorical levels of socio-economic characteristics. In research conducted in Rio Grande do Sul, Brazil, Kato et al. [23] apply hierarchical Bayesian procedures to study ecological correlations of IMR with other development measures. Moreover, Chin et al. [24] estimate a survival regression model that allows for spatially correlated random effects in Nepal. Controlling for individual and community-level covariates, their residual still shows a spatial pattern, indicating that health policies should be locally targeted. These studies highlight the advantages of adopting a space-time modeling framework in the analysis of IMR, and motivate the use of spatial panel data models for our study of Brazilian municipalities between 1980 and 2000, as described next.

### Methods: spatial panel data models

In the present paper, a spatiotemporal modeling approach in the form of a spatial panel structure is adopted. According to Elhorst [25,26], spatial panel data models are more informative, contain more variation and less collinearity among variables than cross-sectional or time series data models. They also increase the degrees of freedom available, resulting in higher efficiency. However, just like their cross-sectional counterparts, the problem of spatial effects, particularly autocorrelation or lack of independence, can arise when data are georeferenced [14,27].

The basic form of the panel data model is as follows:

(1) $$Y_{t}=\alpha +X_{t}\beta +\varepsilon_{t}$$

where *Y_t _*is the variable to be explained (in our case, the IMR in each municipality in each year), *X_t _*is the group of exogenous characteristics (average income, inequality, health care institutions, among others), *α *is the constant term, *β *is the vector of parameters that express the relations between *X_t _*and *Y_t_*, and all the observations and residuals are indexed by location (*i*) and time (*t*). Spatial effects can be incorporated in different ways, considering the fact that each unit *i *is a region. In the case of spatial heterogeneity, one possibility is to use spatial fixed effects whereby a dummy variable is introduced for each spatial unit:

(2) $$Y_{t}=X_{t}\beta +\mu +\varepsilon_{t}$$

Alternatively, random effects can be incorporated by treating the region-specific intercept as a random variable i.i.d., independent of *ε_t_*:

(3) $$\mu \sim N\left(0,\sigma^{2}I_{N}\right)$$

where *μ *is the spatial specific effect that captures the heterogeneity of the municipalities. However, as noted by Elhorst [25], a model with random effects may not be an appropriate specification when it is implemented for a set of irregular spatial units (for instance, municipalities in a country). This is so because the population is sampled exhaustively in this case, creating difficulties to achieve asymptotic results.

Spatial dependence can be incorporated in two distinct forms, as appropriate. One possibility is to allow the errors to display spatial autocorrelation resulting from relevant but omitted variables that follow a spatial pattern. Alternatively, a spatially lagged variable can be introduced, so that the outcome variable at one location is affected by outcomes at neighboring regions. This could be used for instance to represent contagion.

The spatial error autocorrelation model (SEA) is defined as follows:

(4) $$Y_{t}=\alpha +X_{t}\beta +\mu +\varphi_{t},\varphi_{it}=\rho \text{W}\varphi_{\text{it}}+\varepsilon_{it},E\left(\varepsilon_{t}\right)=0,E\left(\varepsilon_{t}^{\prime }\varepsilon_{t}\right)=\sigma^{2}I_{N}$$

where *μ *is a matrix with fixed effects for each region, *φ_t _*is a spatially autocorrelated error term, *W *is a spatial weight matrix that codifies relations of proximity between spatial units, *I_N _*is an identity matrix of order *N *and *ε_t _*is a random (white noise) term. The spatial autocorrelation coefficient *ρ *multiplies the spatial error term *φ*. This term, being a residual, does not require an underlining theoretical model for a spatial or social interaction process.

The estimation of the fixed effects model in the standard way requires the elimination of *α *and *μ *by de-trending the original equation using its averaged counterpart over time. These two elements can be recovered afterwards using other transformations of the original equation (see Baltagi [27], pp. 14-15, and Wooldridge [28], pp. 265-269). The parameters in the demeaned equation are estimated using maximum likelihood, with an iterative two-stage procedure in the case of the spatial error model.

The spatially autoregressive (or lag) model (SAR) is formulated in the following way:

(5) $$Y_{t}=\delta WY_{t}+X_{t}\beta +\mu +\varepsilon_{t},E\left(\varepsilon_{t}\right)=0,E\left(\varepsilon_{t}^{\prime }\varepsilon_{t}\right)=\sigma^{2}I_{N}$$

where, again, *ε_it _*is a random (white noise) term, and the estimation follows a maximum likelihood procedure. The spatial autoregressive coefficient *δ *multiplies the spatially lagged dependent variable, representing the situation where the dependent variable observed for the unit of analysis is jointly determined with that of its neighbors.

Model selection can be conducted based on statistical criteria, such as the goodness of fit and adherence to underlying assumptions, or based on theoretical considerations. After estimation of a pooled and a spatial fixed effects model, spatial dependence tests can be applied (i.e. Lagrange Multiplier-lag and Lagrange Multiplier-error) to determine whether estimation of a spatial model is warranted. The SEA and SAR models can be compared as a robustness check.

The advantage of employing a panel structure is that it enables us to analyze a phenomenon that happens over time in the municipalities of a country. The inclusion of spatial fixed effects is the first step to account for local heterogeneity,^2 ^but is not enough to deal with underlying spatial processes. Therefore, spatial dependence structures can be included to address this issue and provide unbiased and efficient estimators. Not accounting for them may lead to inconsistent estimators.

In order to investigate the temporal trend of the underlying process, it is also possible to develop a complementary model where the dependent variable is the change in the level of IMR, and the independent variables are temporally lagged. One limitation of this form of analysis is that it focuses only on the average effect of each explanatory variable for the IMR level.

### Data

#### Sources

The scope of our analysis is defined by data availability for the desired period and level of regional disaggregation. The main sources of information are the 1980, 1991 and 2000 Brazilian Census of Population (Censo Demográfico, Instituto Brasileiro de Geografia e Estatística - IBGE). All sources of data used in this work are publicly available and can be obtained from the websites of IBGE, the Ministry of Health and Ipeadata.

Data are obtained for three separate years covering a period of two decades. In 1980, the sample consists of 25 percent of the population. Both in 1991 and in 2000, the samples are based on a 10 percent basis for localities with more than 15,000 inhabitants and a 20 percent base otherwise. The variables obtained from these sources refer to income, inequality, education, size of the population and other characteristics of the areas of analysis.

Health infrastructure information is derived using data obtained from the Medical and Sanitary Assistance Surveys, of 1981, 1990 and 1999.^3 ^This is an annual survey conducted by the Brazilian Ministry of Health, from which we obtain information on health care institutions, as proxies for health care provision. More details on the variables can be found in Table 1, also including the expected sign in relation with IMR.

**Table 1:** Definition of variables, source, reference years and expected signal

Variable name	Code	Description	Source	Reference years	Expected signal
**Infant Mortality Rate**	*imr*	Number of people that will not complete 1 year of life from 1,000 born alive in the reference year.	IPEAdata, UNDP (Humand Development Atlas of Brazil)	1980, 1991 and 2000	

**log(Intant Mortality Rate)**	*l_imr*	Logarithm of the Infant Mortality Rate.	IPEAdata, UNDP (Humand Development Atlas of Brazil)	1980, 1991 and 2000	Dependent

**Number of public health care institutions per 1,000 people**	*pub_hospit*	Number of public health care institutions in the MCA divided by the population and multiplied by 1,000.	DATASUS - Ministry of Health	1981, 1990 and 1999*	**-**

**Number of private health care institutions per 1,000 people**	*priv_hospit*	Number of private health care institutions in the MCA divided by the population and multiplied by 1,000.	DATASUS - Ministry of Health	1981, 1990 and 1999*	**-**

**% of households with access to water**	*water access*	Percentage of households with access to water.	Microdata of the Demographic Census (IBGE)	1980, 1991 and 2000	**-**

**% of households with access to sanitation**	*sanitation*	Percentage of households with access to sanitation.	Microdata of the Demographic Census (IBGE)	1980, 1991 and 2000	**-**

**Average income (2000 R$)**	*average income*	Average household per capita income.	Microdata of the Demographic Census (IBGE)	1980, 1991 and 2000	**-**

**Gini Index**	*gini*	Gini index (ranges from 0 - complete equality - to 1 - only one person concentrates the income), obtained from the per capita household income.	Microdata of the Demographic Census (IBGE)	1980, 1991 and 2000	**+/-**

**Female illiteracy rate (15 years old or more)**	*fem_illiteracy*	Number of women older than 15 years that are illiterate over the total of women older than 15.	Microdata of the Demographic Census (IBGE)	1980, 1991 and 2000	

**% of urban population**	*%urban_pop*	Percentage of people living in the urban area of the MCA.	Microdata of the Demographic Census (IBGE)	1980, 1991 and 2000	**-**

**Adolescent fertility rate (10-19 years old)**	*adol_fertility*	Percentage of women from 10-19 years old with children.	Microdata of the Demographic Census (IBGE)	1980, 1991 and 2000	**+**

W * Number of public health care institutions per 1,000 people	W*pub_hospit	Spatial lag (average of the neighbors) of the number of public health care instituitions over 1,000 people.	DATASUS - Ministry of Health	1981, 1990 and 1999*	**-**

W * Number of private health care institutions per 1,000 people	W*priv_hospit	Spatial lag (average of the neighbors) of the number of private health care instituitions over 1,000 people.	DATASUS - Ministry of Health	1981, 1990 and 1999*	**-**

W * % of households with access to water	W*water access	Spatial lag (average of the neighbors) of the percentage of households with access to water.	Microdata of the Demographic Census (IBGE)	1980, 1991 and 2000	**-**

W * % of households with access to sanitation	W*sanitation	Spatial lag (average of the neighbors) of the percentage of households with access to sanitation.	Microdata of the Demographic Census (IBGE)	1980, 1991 and 2000	**-**

*with respect to population in 1980, 1991 and 2000, respectively.

An usual limitation in works that deal with infant mortality in Brazil is the quality of data, which is related to the underreporting of births and deaths in some parts of the country (mainly the North and the Northeast, [29]). In the present study we do not face such limitation, since calculation of IMR data made available by the UNDP is based on census information [30], specifically the question about how many children each mother had who did not survive their first year of life.

With regards to the unit of analysis, it is important to note that between 1980 and 2000 new municipalities were created as a consequence of the partition or merging of previously existing municipalities. In order to organize the panel, it is important to define areas that remain spatially compatible throughout the period covered by the study. In order to address this issue, we use the Minimum Comparable Areas created by the Applied Institute of Economic Research (IPEA) for the purpose of making official statistics comparable between census years. These areas guarantee that each unit of analysis has the same size in every period; then, it is possible to consider both merges and splits of municipalities along time [31].

### Infant mortality data

Health outcomes can be defined in several ways. Victora et al. [16] and Hanmer et al. [2] list those relating to mortality: neonatal mortality (from the first to the twenty-seventh day of life), post-neonatal mortality (from the twenty-eightieth day to one year of life), child mortality (from the first to the fifth year of life), under-five mortality (number of deaths of children less than five years old), and finally, infant mortality (children under one year old).

In this study, we concentrate on infant mortality, as it is readily available from data provided by UNDP (Human Development Atlas of Brazil) in a regionally disaggregated form. It is also one of the measures most affected by precarious life conditions, and depends less on other factors such as on congenital diseases (as the neonatal) or vaccination policies (as the under-five mortality rate). Even though it would be interesting to assess the different causes of neonatal and post-neonatal infant mortality rates [32], the availability of data restricts this work to the under-one year of life mortality rate.

**Figure 1:**
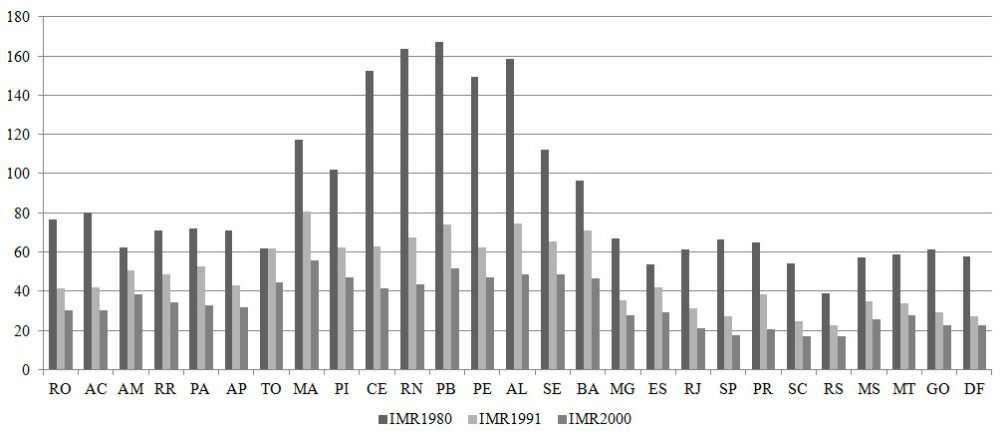
**Infant mortality rate (IMR) across Brazilian states, 1980, 1991 and 2000**. Source: Ipeadata.

Brazil has shown consistent reduction of infant mortality rates in the last decades, improving from 123.19 deaths per 1,000 live births in 1970 to 85.20 in 1980, 44.68 in 1991, and 30.57 deaths per 1,000 live births in 2000.^4 ^Despite major gains, there remain substantial regional differences in the pattern of reduction, as can be seen in Figure 1. The states of the Northeast of Brazil^5 ^had in 1980 a noticeably higher level of infant mortality than the rest of the country. In spite of substantial reductions, these states still had the highest infant mortality rates in the country in 2000.

**Figure 2:**
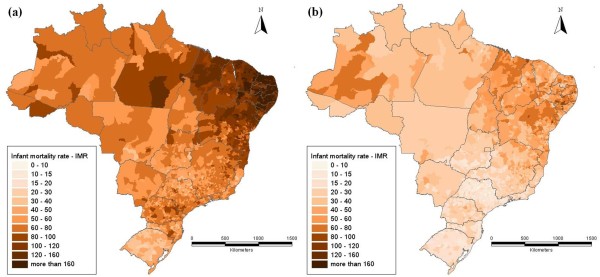
**Infant mortality rate (IMR) distribution in the MCAs in (a) 1980 and (b) 2000**. Source: Ipeadata.

Using the Minimum Comparable Areas (MCA, 3,659 units) it is possible to visualize the changes in the geographical distribution of IMR. The maps presented in Figure 2 show a very distinctive regional pattern, characterized by high levels of IMR concentrated in parts of the Northeast and low levels of IMR in the South. Even though there has been a broad reduction in IMR levels from 1980 to 2000, there still remains glaring regional inequality.

**Figure 3:**
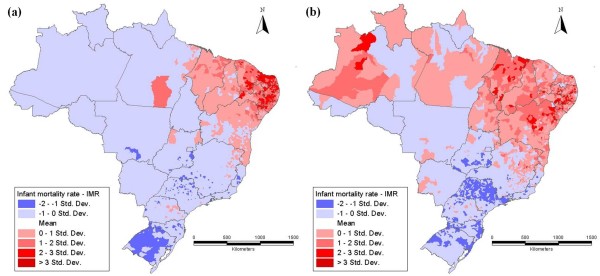
**Standard deviation of the IMR in 1980 (a) and 2000 (b)**. Source: Authors' elaboration over Ipeadata information.

Figure 3 displays the standard deviation of IMR across the country. Clearly, both in 1980 and 2000, the highest values over the average are in the Northeast and the lowest ones are in the Southeast. It is important to note that by 2000 an overall reduction in the infant mortality rate had been achieved, however, relatively high IMR values still prevail in some areas of the country revealing a delay of development. Markedly, the gains in reducing infant mortality have been uneven, and the developmental challenge of improving life conditions remains to be fully addressed.

**Figure 4:**
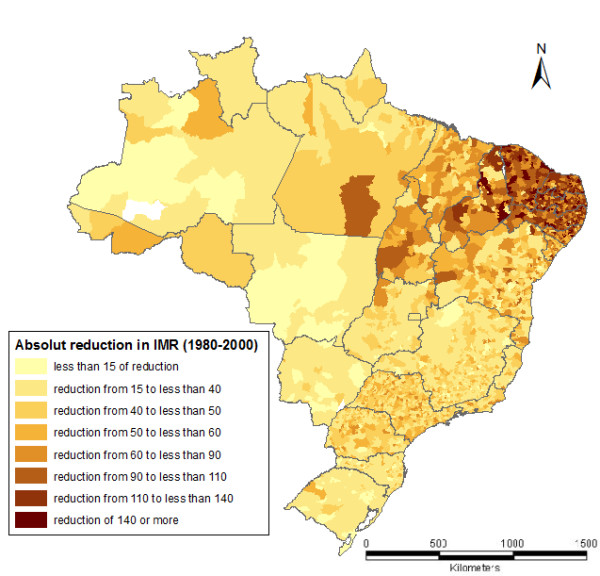
**Percentual reduction of the IMR between 1980 and 2000, by MCAs**.

Substantial differences in the pace of reduction of the IMR around the country can be appreciated from Figure 4. Noticeably, there is a large group of municipalities in the Northeast of the country that showed a significant reduction of their IMR between 1980 and 2000. Many municipalities of this cluster, which displayed the highest IMR levels in 1980 (see Figure 2), actually saw a decrease in IMR of more than 140 deaths/1,000 people born alive.

### Selection of variables and descriptive statistics

**Figure 5:**
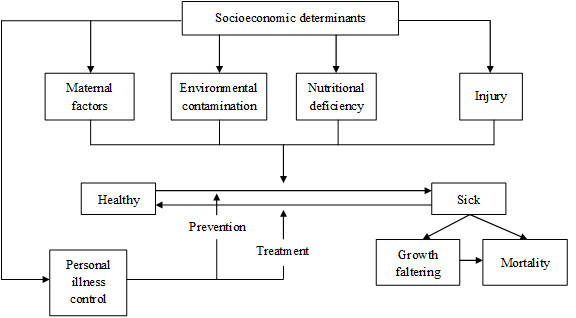
**Operation of the five groups of proximate determinants on the health dynamics of a population**. Source: Adapted from Mosley and Chen (1984).

Selection of variables is informed by results previously reported in the literature and by the conceptual framework proposed by Mosley and Chen [33], and used by Sastry [34] and Hanmer et al. [2] (see Figure 5). According to this conceptual framework, a set of proximate determinants can be used to assess the effect of indirect determinants. In this section we discuss the variables selected for the analysis. The descriptive statistics of these variables appear in Table 2.

**Table 2:** Descriptive statistics of the variables considered in the analysis

	Average			Median			Standard Deviation			Maximum			Minimum		
	**1980**	**1991**	**2000**	**1980**	**1991**	**2000**	**1980**	**1991**	**2000**	**1980**	**1991**	**2000**	**1980**	**1991**	**2000**

*imr*	86.77	49.22	33.73	69.37	41.73	29.31	45.23	24.39	18.08	257.89	125.24	98.12	22.19	11.08	5.38

*l_imr*	1.89	1.64	1.46	1.84	1.62	1.47	0.21	0.22	0.24	2.41	2.10	1.99	1.35	1.04	0.73

*pub_hospit*	0.22	0.34	0.40	0.17	0.27	0.34	0.19	0.25	0.27	1.83	2.28	2.87	0.00	0.00	0.00

*priv_hospit*	0.05	0.10	0.08	0.03	0.08	0.05	0.07	0.10	0.10	1.09	0.79	0.87	0.00	0.00	0.00

*water access*	0.24	0.43	0.63	0.19	0.41	0.66	0.22	0.24	0.21	0.99	1.00	1.00	0.00	0.00	0.00

*sanitation*	0.11	0.18	0.30	0.00	0.00	0.18	0.19	0.27	0.31	0.92	0.98	0.99	0.00	0.00	0.00

*average income*	141.78	126.67	188.59	125.12	109.02	177.14	81.23	77.00	109.49	614.64	717.73	892.75	10.96	24.87	35.37

*gini*	0.49	0.53	0.55	0.49	0.53	0.55	0.07	0.06	0.07	0.86	0.92	0.80	0.27	0.32	0.30

*fem_illiteracy*	0.38	0.30	0.20	0.35	0.26	0.18	0.16	0.15	0.11	0.91	0.83	0.54	0.03	0.02	0.02

*%urban_pop*	0.44	0.55	0.63	0.40	0.54	0.64	0.23	0.23	0.21	1.00	1.00	1.00	0.02	0.02	0.07

*adol_fertility*	0.05	0.06	0.08	0.05	0.05	0.08	0.02	0.03	0.03	0.20	0.21	0.26	0.00	0.00	0.00

W*pub_hospit	0.21	0.32	0.38	0.19	0.29	0.36	0.11	0.14	0.16	0.96	1.40	1.67	0.00	0.00	0.00

W*priv_hospit	0.06	0.10	0.09	0.05	0.09	0.08	0.04	0.06	0.06	0.31	0.45	0.49	0.00	0.00	0.00

W*water access	0.26	0.44	0.64	0.21	0.42	0.65	0.18	0.20	0.16	0.97	0.97	0.99	0.00	0.00	0.00

W*sanitation	0.12	0.19	0.31	0.02	0.05	0.21	0.17	0.25	0.27	0.84	0.95	0.96	0.00	0.00	0.00

One of the most commonly used factors to explain the pattern of IMR is the supply of health services. Sastry [34], Leal and Szwarcwald [17], Hanmer et al. [2] and Macinko et al. [35], among others, highlight the relevance of providing health care services in past reductions of infant mortality in the Brazilian context.

Health can be considered a public good or at least a semipublic good, in the sense that it has a high level of externalities (the benefits that come from its consumption are not totally internalized by the individual who purchases it). Besides, the government provision has also a social role. When the prices of health services are defined in the market, there may be a significant part of the population that will be excluded from these services. This can be prejudicial to the survival of the individuals, justifying public provision [36,37].

In the Brazilian context, Baer et al. [38] highlight that public provision of health services was not directed to the poorer class, at least until the 1990's. Highly specialized services were often offered to and accessed by high and middle income individuals. At the same time, basic services supply was neglected, resulting in congestion and low quality provision. Therefore, poor people often had to resort to relatively costly private services, or to go without access to health care at all.

Health infrastructure has increased steadily in the decades studied, however characterized by the under-provision of public health care. According to data from IBGE (*Pesquisa de Assistência Médica e Sanitária*), less than 30% of hospital beds were in public institutions in 1999. Consequently, when comparing public and private health care provision, a higher explanatory power of private institutions in the reduction of the IMR among Brazilian municipalities is expected.

**Figure 6:**
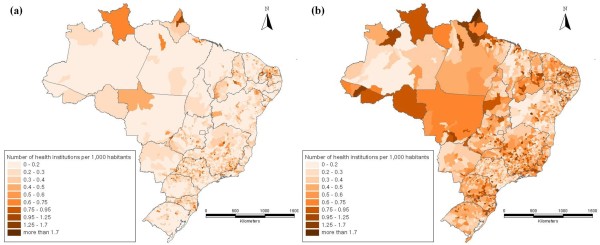
**Health care institutions per 1,000 habitants, (a) 1981 and (b) 1999**. Source: DataSUS.

**Figure 7:**
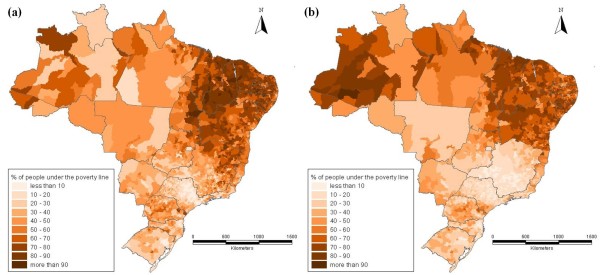
**Poverty rate (% of the population under the poverty line - R$ 75.50 in values of 2000), (a) 1980 and (b) 2000**. Source: Micro-data of the Demographic Census, IBGE.

Figure 6 shows that the spatial distribution of health care institutions does not seem to have a similar regional pattern as the distribution of the IMR. It is important to determine which other variables explain the regional inequality of the IMR in Brazil. Socio-economic variables are considered important, as many studies have found significant relationship between the improvement of social conditions and the reduction of infant mortality [2,8,17,18]. This can be seen in Figure 7, where the distribution of the poverty rate is compared between 1980 and 2000. The similarity with the distribution of poverty rates is more marked than in the case of health infrastructure. This socio-economic aspect can be taken into account by including both the average income and income inequality.

Other forms of infrastructure that may have an impact on IMR include sanitation and piped water provision. Access to these services allows families to improve their hygiene and therefore develop fewer diseases [21,39]. Provision of these services is directly associated to the degree of urbanization of the municipality. In addition, there are other advantages of being in an urban area that may allow the family to achieve better life conditions, such as, for instance, having access to garbage collection services. Overall, there were increases in sanitation provision between 1980 and 2000. However, penetration rates remained low, and more limited than clean water coverage.

In terms of socio-economic and demographic variables, Deaton [40] proposes a theoretical framework in which progressive income redistribution would result in improved health measures following decreasing returns of income over health outcomes. Therefore, inequality reduction should be expected to decrease infant mortality rates. Average income increased 33% between 1980 and 2000; however, the average inequality in the Minimum Comparable Areas (measured by means of the Gini index) grew considerably in the period considered (from 0.49 to 0.55).

Another important variable that can be included in the set of socio-economic characteristics is the level of education of the parents. In general, improving the education level of the population is found to exert a strong effect over the measures that families adopt to avoid basic health problems (see Soares [39] and Alves and Belluzzo [18]).

Adolescent fertility ratio can be included as a measure of family structure, although past research has not always found a significant result for this variable [12]. Mixed findings in the literature are usually attributed to the fact that the proportion of under-15 mothers is very low in the international context. Therefore, while teen mothers may represent a higher risk to their infants, the percentage of adolescent mothers may be too small to have a significant effect over the IMR.

Finally, we also consider the spatial lag of independent variables for inclusion in the spatial panel model. The ones included in this analysis relate to regionally available health care services, water and sanitation facilities provision and the urban size of the neighbors, beyond that of the spatial unit itself. In the case of urban population, for example, the spatial lag is an indicator of the degree of urbanization in neighboring areas. Therefore, the spatial lag is a simple average of the values of these variables in the contiguous spatial units. In this sense, spatially lagged variables represent relevant characteristics of the neighbors that can affect the infant mortality rate of the unit of analysis.

## Results and discussion

Estimation of the models reported in this section was conducted using LeSage's MATLAB routines, which are available online^6 ^and incorporate efficient treatments for situations with a large number of spatial units [25]. Some of these routines have been recently updated by Elhorst [41].

The results of models considering the variables mentioned before are displayed in Table 3 and Table 4. In order to reduce the scale of the dependent variable, IMR are transformed using the natural logarithm operator. This transformation also ensures that estimated values are not negative when converted back to the original scale. For comparative purposes, two models are presented in Table 3, namely a pooled model (i.e. it does not consider regional fixed effects) and a fixed effects panel model.

**Table 3:** Results for the pooled and the spatial fixed effects models for the log of infant mortality rate

Dependent variable: log(infant mortality rate)	Pooled model		Fixed Effects	
**Constant**	4.064	***		

**Number of public health care institutions per 1,000 people**	0.004		-0.011	

**Number of private health care institutions per 1,000 people**	-0.153	***	-0.246	***

**% of households with access to water**	0.151	***	-0.058	**

**% of households with access to sanitation**	-0.040		-0.214	***

**Average income (2000 R$)**	-0.001	***	0.000	

**Gini Index**	-0.103	**	-0.109	

**Female illiteracy rate (15 years old or more)**	1.805	***	0.697	***

**% of urban population**	-0.150	***	-0.770	***

**Adolescent fertility rate (10-19 years old)**	-0.983	***	0.408	***

W * Number of public health care institutions per 1,000 People	-0.070	***	-0.208	***

W * Number of private health care institutions per 1,000 people	-1.216	***	-1.286	***

W * % of households with access to water	-0.908	***	-0.900	***

W * % of households with access to sanitation	-0.064	**	-0.747	***

**R**^**2**^	0.7311		0.7906	

**Adjusted R**^**2**^	0.7308		0.7904	

**$\sigma_{\varepsilon }^{2}$**	0.1137		0.0401	

**LM test - spatial lag**	0.58		9,708.69	***

**robust LM test - spatial lag**	0.07		1,710.14	***

**LM test - spatial error**	221.95	***	8,090.69	***

**robust LM test - spatial error**	221.43	***	92.14	***

**N**	10,977		10,977	

* α = 0.10; ** α = 0.05; *** α = 0.01;

**Table 4:** Results for the fixed effects spatial error model (SEA) and the fixed effects spatial autocorrelation model (SAR) for the log of infant mortality rate

Spatial fixed effects				
**Dependent variable: log(infant mortality rate)**	**FE SEA model**		**FE SAR model**	

**Number of public health care institutions per 1,000 people**	-0.048	***	-0.012	

**Number of private health care institutions per 1,000 people**	-0.166	***	-0.159	***

**% of households with access to water**	-0.259	***	-0.142	***

**% of households with access to sanitation**	-0.222	***	-0.160	***

**Average income (2000 R$)**	-0.001	***	0.000	***

**Gini Index**	0.244	***	0.083	***

**Female illiteracy rate (15 years old or more)**	0.187	***	0.305	***

**% of urban population**	-0.047	*	-0.221	***

**Adolescent fertility rate (10-19 years old)**	0.168	***	0.139	*

W * Number of public health care institutions per 1,000 people	-0.213	***		

W * Number of private health care institutions per 1,000 people	-0.561	***		

W * % of households with access to water	-1.000	***		

W * % of households with access to sanitation	-0.569	***		

**atial error autocorrelation (ρ)**	0.786	***		

**Spatial lag of the log(infant mortality rate) (δ)**			0.7789	***

**R**^**2**^	0.8875		0.9554	

**$\sigma_{\varepsilon }^{2}$**	0.0194		0.0188	

**Loglikelihood**	5,264.64		5,447.57	

**AIC**	-10,501.28		-10,875.14	

**Corr-squared**	0.7699		0.8045	

**N**	10,977		10,977	

* α = 0.10; ** α = 0.05; *** α = 0.01;

As seen in Table 1, the pooled model gives the lowest coefficient of determination R^2 ^and suffers from several deficiencies. In this model, both sanitation and public health institutions are not significant. Also, the sign of water access is counterintuitive. The adolescent fertility rate has a negative sign. The strongest effects over the IMR are found with the female illiteracy rate (1.8) and the spatially lagged number of private health care institutions (-1.2).

The panel with fixed effects leads to some changes in the main results compared to the pooled one. Now, sanitation is highly significant and has the expected sign. However, average income and income inequality lose their significance. The coefficient of illiteracy rate is three times smaller than the one in the pooled framework, and the variable measuring public health care now has the expected sign, even if it is still not significant. Spatially lagged variables are all significant and have a strong effect over the IMR, suggesting that there is a spatial spillover in local health and infrastructure policies.

It is important to notice, however, that the spatial dependence tests performed for both models indicate the presence of significant spatial dependence, in the form of spatial error autocorrelation or a spatially lagged dependent variable. Spatial dependence is a serious issue that leads to inefficient and/or biased coefficients. Therefore, in Table 4 we present the results of the estimation of the models accounting for spatial effects.

Two models are estimated that explicitly consider, in addition to the spatially lagged independent variables, spatial error autocorrelation (SEA) and spatial autocorrelation of the dependent variable (SAR), respectively. A general observation is that these models provide better goodness of fit indicators. Furthermore, examination of the residuals indicates that these are homoscedastic. Finally, the spatial effect is highly significant in both cases, and inference becomes more reliable, as the spatial structure corrects for the inconsistency of the coefficients. In spite of their structural differences, all variables have the same sign in the two models, and only the variable for public health care institutions loses its significance in the SAR model. This first aspect means that our model seems to be robust to the form of spatial structure adopted.

As previously mentioned, model selection can be based on statistical or theoretical criteria. In the present case, the SAR model returns a slightly higher *R^2^*. The two models are comparable in terms of their AIC, squared-R, and variance, and therefore, on purely statistical grounds, both seem to provide very similar levels of goodness of fit. Conceptually, our view is that a model with a spatially lagged dependent variable is less appealing, because at the level of aggregation considered in this analysis (municipalities) there is no plausible mechanism to explain why IMR should be high when it is high in neighbouring regions, other than through the operation of common variables or spillovers. For this reason, we concentrate our discussion on the SEA model.

The fixed effects SEA model provides a number of valuable insights. Income is widely acknowledged to have a strong impact on the reduction in IMR. Our analysis, after controlling for a large number of confounding factors, is able to confirm the significance of income, even if the effect observed for this variable is relatively small (-0.001 - which means that an increase of 1 p.p. in average income reduces the level of infant deaths by 0.001 over 1,000 born alive). The results also indicate that as income inequality and women illiteracy rate grow, IMR also tend to increase by factors of 0.245 and 0.185, respectively. In the final analysis, we find that adolescent fertility rate associates positively with IMR (0.168), suggesting that family planning can help to reduce infant mortality.

In terms of health infrastructure, the results indicate that the number of private health care institutions exerts a stronger effect than public institutions (-0.165 and -0.048, respectively). This result aligns with the suggestion that poorer families have to resort to private health care, as specialized public services were often offered to and accessed by high and medium income individuals, and basic public services supply was neglected, resulting in a congested and low quality provision. Consequently, if private health care institutions are offered, the options of treatment for the poor are increased. Furthermore, water access and sanitation have a strong negative impact over the IMR. The first one is almost universally spread in the country, but even nowadays sanitation services cover only around 50% of the Brazilian households.

Finally, spatially lagged variables have almost twice the impact of their local counterparts. Any policy aiming to improve life conditions must take this result into consideration. Hence, there are indirect regional effects of improvements in health care infrastructure or the level of public services access. Further policy recommendations relate to the reduction of income inequality and illiteracy rate, and spreading information regarding fertility control.

While the models reported in Table 4 correlate the levels of IMR to their cotemporaneous variables, they fail to provide a sense of the temporal trends. An alternative perspective can be gleaned by redefining the dependent variable as the first difference between time periods. The independent variables, on the other hand, are for the initial time period, and thus enter exogenously the model. This procedure results in *T *= 2 time periods (1980 to 1991 and 1991 to 2000) and reduces the size of our sample. It is important to note that the objective of this model is to identify the effect of initial conditions over the change in the infant mortality rate. The modeling strategy followed for this analysis mirrors the one described above. Once again, the spatial dependence is identified in the fixed effects model, requiring the estimation of a spatial panel model. For the sake of brevity, only the results of the spatial models (SEA and SAR) are reported in Table 5.

**Table 5:** Results for the fixed effects spatial error model (SEA) and the fixed effects spatial autocorrelation model (SAR)

Spatial fixed effects				
**Dependent variable:** **log(infant mortality rate)_t _- log(infant mortality rate)_t-1_**	**FE SEA model**		**FE SAR model**	

**Number of public health care institutions per 1,000 people_t-1_**	0.046	**	0.033	*

**Number of private health care institutions per 1,000 people_t-1_**	0.182	***	0.184	***

**% of households with access to water_t-1_**	0.212	***	0.170	***

**% of households with access to sanitation_t-1_**	0.133	***	0.134	***

**Average income (2000 R$)_t-1_**	0.000		0.000	

**Gini Index_t-1_**	-0.248	***	-0.264	***

**Female illiteracy rate (15 years old or more)_t-1_**	-0.275	***	-0.346	***

**% of urban population_t-1_**	-0.061		-0.069	

**Adolescent fertility rate (10-19 years old)_t-1_**	-0.385	***	-0.222	

W * Number of public health care institutions per 1,000 people_t- 1_	-0.069			

W * Number of private health care institutions per 1,000 people_t-1_	0.593	***		

W * % of households with access to water_t-1_	0.189	***		

W * % of households with access to sanitation_t-1_	0.204	***		

**Spatial error autocorrelation (ρ)**	0.553	***		

**Spatial lag of the log(infant mortality rate) (δ)**			0.5530	***

**R**^**2**^	0.5478		0.6607	

**$\sigma_{\varepsilon }^{2}$**	0.0262		0.0262	

**Loglikelihood**	2,724		2,723	

**AIC**	-5,421		-5,426	

**Corr-squared**	0.1792		0.1803	

**N**	7,318		7,318	

* α = 0.10; ** α = 0.05; *** α = 0.01;

In order to interpret the coefficients of the model, it bears noting that a negative value of the dependent variable means that the IMR, as was the case countrywide, decreased in the implied period of time. The signs of the coefficients are the opposite of the models reported in Table 4. This provides an interesting, and intuitive, insight. For instance, a positive sign for the number of health care institutions in the previous period of time means that the dependent variable is *less *negative as this variable increases. Negative values for the coefficients associated with illiteracy rates and adolescent fertility rates imply *more *negative values of the difference, and therefore greater gains in IMR. Clearly, since IMR is a zero-bounded variable, the trend indicates that greater gains were obtained in regions with the worst initial conditions (e.g. lower levels of infrastructure provision, higher levels of illiteracy). The implication therefore is that further gains in IMR become increasingly difficult as conditions improve. This apparently simple conclusion nonetheless suggests that allocation of resources should try to efficiently target programs with potential to generate greater reductions of IMR.

## Conclusions

Infant mortality is an indicator of considerable policy interest from the perspective of developmental goals. As a measure of development, it is an essential indicator of the freedom of choice that people will have as they manage to survive to their first years of life. The objective of this paper has been to investigate the factors that influence the variation of IMR in Brazil in the period 1980-2000. Analysis was based on municipal-level data (Minimum Comparable Areas) and a wide array of infrastructure, socio-economic, and demographic information. In recognition of the importance of spatial effects when modeling georeferenced information, we adopted a spatial panel data analysis methodology.

The following original findings are reported in this paper.

In terms of the methods, we estimated four models: a pooled model, a panel model with fixed spatial effects, and two panel models with spatial fixed effects and spatial dependence, SEA and SAR. The results suggest that ignoring spatial effects in the analysis can lead to misleading inference caused by inefficient and/or biased coefficients. This implication is important, since to our knowledge there are only a few works considering space explicitly in such a framework.

More substantively, the results of the fixed effects Spatial Error Model indicate that IMR reduction in Brazil over the two decades studied related to socio-economic characteristics and the provision of infrastructure. The relevance of some of these variables has been previously reported in the literature. In addition to this, we find that the spatial dimension of several policy variables is essential, as there are spatial spillovers related to health care infrastructure, water, and sanitation. This suggests that the provision of infrastructure on a geographically broad basis tends to induce self-reinforcing effects whose impact could be dampened under centralization of services. The main policy implications are robust to a change in the spatial dependence structure, represented here by a fixed effects SAR model. Finally, we find that reductions in IMR over the period analyzed tended to be smaller in places that had higher starting levels of infrastructure and lower levels of illiteracy.

A possible limitation to this work may be the presence of endogeneity in the models presented in Table 4. This would be the case if some variables are jointly determined (for instance, if low income causes high IMR and in turn high IMR depresses the economy). The models in Table 5 which account for temporal trends and timely lagged independent variables are less subject to endogeneity issues, especially given the ten years lag used in the analysis.

With these original findings, the paper contributes to expand our understanding of the factors that influence IMR in the particular case of Brazil and in developing countries in general.

## Endnotes

^1 ^International institutions such as the United Nations (UN), the Organization for Economic Cooperation and Development (OECD), and the World Bank have adopted this perspective. The Millennium Development Goals, set forth by the UN, are a clear example of this view, with three of eight stated goals being related to health issues.

^2 ^Its counterpart, the random effects model, cannot be applied because of the structure of spatial data.

^3 ^Information available at http://www.datasus.gov.br (last accessed in 11/15/2011).

^4 ^Information obtained within http://www.ipeadata.gov.br

^5 ^Maranhão (MA), Piauí (PI), Ceará (CE), Rio Grande do Norte (RN), Paraíba (PB), Pernambuco (PE), Alagoas (AL), Sergipe (SE) and Bahia (BA).

^6 ^http://www.spatial-econometrics.com/ - in order to run the routines with a large number of spatial units using the algebraic method instead of using the numeric method, *Nhes *was changed from 500 to 4.000.

## Competing interests

The authors declare that they have no competing interests.

## Authors' contributions

All authors contributed equally to the conceptual framework for this study. Statistical analysis was conducted by AMB under the supervision of EH and AP. All authors contributed to the discussion and interpretation of results. The final manuscript was read and approved by all authors.

## Pre-publication history

The pre-publication history for this paper can be accessed here:

http://www.biomedcentral.com/1471-2458/12/181/prepub
